# There is only one winner: The negative impact of red deer density on roe deer numbers and distribution in the Słowiński National Park and its vicinity

**DOI:** 10.1002/ece3.7538

**Published:** 2021-05-18

**Authors:** Jakub Borkowski, Rafał Banul, Jolanta Jurkiewicz‐Azab, Czesław Hołdyński, Justyna Święczkowska, Maciej Nasiadko, Dariusz Załuski

**Affiliations:** ^1^ Department of Forestry and Forest Ecology University of Warmia and Mazury Olsztyn Poland; ^2^ Słowiński National Park Smołdzino Poland; ^3^ Wildlife Monitoring Project Mogilany Poland; ^4^ Department of Botany and Nature Conservation University of Warmia and Mazury Olsztyn Poland; ^5^ Vita Arbor Maciej Nasiadko Szczytno Poland; ^6^ Department of Genetics, Plant Breeding and Bioresource Engineering University of Warmia and Mazury Olsztyn Poland

**Keywords:** interspecific competition, population density, protected areas, red deer, roe deer, ungulate assemblage

## Abstract

Red and roe deer are the most numerous cervids in Europe, and they occur in sympatry in most regions. Roe deer were considered to be an inferior competitor in studies in which they co‐occurred with fallow deer or muntjac. Despite the remarkable overlap of their ranges, there are few studies on the competition between the red and roe deer. Since interspecific interactions among ungulates are often related to their mutual densities, the current study focused on the effects of high red deer density on the roe deer numbers and spatial distribution in the unhunted Słowiński National Park (SNP) in northern Poland and forest districts open to hunting bordering the park. Using fecal pellet group counts, it was found that in the forest districts (where red deer densities were 2–3 times lower than in the SNP), roe deer densities were significantly higher than in the park. The red‐to‐roe deer density ratio was 10.8 and 2.7, in the SNP and the surrounding forest districts, respectively. Moreover, in the SNP, the roe deer distribution was negatively affected by the red deer habitat use, while in the hunting areas, such an effect was not recorded. The negative influence of the red deer on the roe deer population in the park was most probably due to the red deer impact on food availability. The biomass of the plant groups forming the staple food of the roe deer (*Rubus* spp., forbs, dwarf shrubs) was significantly higher in the fenced plots than in the unfenced ones. Lack of hunting in the protected areas may benefit only some species in ungulate assemblages which, in turn, may contradict one of their objectives—to maintain viable and ecologically functional populations.

## Graphical abstract

The manuscript describes (as one of the first examples) the negative impact of high red deer density on roe deer population. The impact is through resource competition. It is worth to mention that both species are, except for wild boar, the most numerous large herbivore species in Europe.

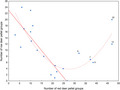

## 1 INTRODUCTION

Interactions between species in large herbivore assemblages are a key factor in understanding the processes shaping their composition (Arsenault & Owen‐Smith, 2002; Murray & Illius, 2000) as well as their impact on ecosystems (Cumming & Cumming, 2003; Latham et al., 1996). In the majority of the large herbivore assemblages, relative densities are strongly influenced by hunting (Apollonio et al., 2017), which modifies the species composition, interspecific processes, and impact on the habitats. Protected areas are not only a fundamental issue in nature conservation (Naughton‐Treves et al., 2005; Watson et al., 2014) but also, due to the lack of hunting, they are often places where large herbivore numbers tend to increase in comparison with the unprotected surroundings (Porter & Underwood, 1999; Wagner, 2006). This increase has been recorded not only in large protected areas like Yellowstone National Park (Wagner, 2006) but also in much smaller parks (Borkowski et al., 2019) such as the majority of European national parks.

A significant issue in the protected areas is the restoration of ecological patterns and processes that have been affected by anthropogenic disruption (Hayward, 2009; Suding et al., 2004) including the restoration of ungulate assemblages (Venter et al., 2014). As already mentioned, a lack of population control can lead to an increase in ungulate density. In terms of high density/limited resources, interspecific competition between the members of the community is likely to occur with the possible exclusion of some of them (Wiens, 1993). Thus, an important issue is to what extent the lack of hunting positively influences all species involved versus those which are better competitors. In many developed countries, ungulate, especially deer populations, occur in high densities also beyond the protected areas, which has been recorded both in Europe (Apollonio et al., 2010) and in North America (Côté et al., 2004). Therefore, competition (as well as other interspecific interactions) seems to be an important research direction.

Roe (*Capreolus capreouls*) and red (*Cervus elaphus*) deer belong to the most numerous large herbivore species in Europe (Apollonio et al., 2010), and in many places, they occur in sympatry. Roe deer have been considered to be a species susceptible to competition from other ungulates (Latham, 1999) and it has been demonstrated that roe deer populations may be under the negative influence of muntjac (*Muntiacus reevesi*) (Hemami et al., 2005) or fallow deer (*Dama dama* *)*(Ferretti et al., 2011; Focardi et al., 2006). Information on the relations between red and roe deer is rather scarce. Latham et al. (1996) found a negative correlation between red and roe deer numbers in 20 different forests in Scotland. On a small scale (single area), Richard et al. (2010) found that red deer density negatively influences the body mass of roe deer fawns, also suggesting interspecific competition. To the best of the authors’ knowledge, there is only one paper documenting the negative red deer influence on roe deer distribution on a small scale (Torres et al., 2012); however, it concerns the southern limit of both species distribution, where they occur in low densities. Information on such an impact from more representative ecological conditions of Europe is lacking. In general, the competition between two different species can be twofold (Birch, 1957): interference competition (including adverse social interactions) and resource competition (when species compete for a shared resource of food or space, alternatively—one species depletes a resource and limits its availability to the other species). Roe deer are highly selective feeders, while the red deer food niche is remarkably broader (Storms et al., 2008). As a result, red deer can consume all of the items found in the roe deer diet, and therefore, at a high red deer density, they would be expected to negatively affect the roe deer population, especially in the winter when resources are limited (Latham et al., 1999).

In a previous paper (Borkowski et al., 2019), it was demonstrated that the red deer density in the nonhunted Słowiński National Park is high (c.a. 26 ind./100 ha of the forest areas) and 2‐ to 3‐fold higher than that recorded in the adjacent forest hunting areas. The present paper reports the results of a study concerning roe deer numbers, their spatial relations with the red deer, and the availability of food resources. It is hypothesized that roe deer numbers, spatial distribution, and food resources will be negatively influenced by the red deer population. It is predicted (P 1) that the roe deer density in the Słowiński National Park will be lower than that of the red deer. At the same time, it is predicted (P 2) that despite a lack of hunting/population control in the park, roe deer density in the protected area will be lower than that in the managed forest districts where hunting is allowed. The next prediction (P 3) is that the spatial distribution of the roe deer population in the park will be negatively affected by the red deer distribution, while in the forest districts, due to the lower red deer density, such a relationship will not exist.

Although the roe deer is basically a woodland species (Hewison et al., 1998), it often selects woodland edges (Lovari et al., 2017), and therefore, it is predicted (P 4) that the roe deer distribution in the forest area of the park will be under the influence of the distance to nonforest habitats. The effect of the distance to the forest boundaries with open habitats (arable grounds, meadows) is predicted to be negative. However, due to the small body size of the roe deer, the effect of the distance to forest boundaries with wetlands (swamps and reeds) will be positive, which is caused by the high water level and consequent relative inaccessibility of the habitats to the roe deer. It was also predicted (P 5) that in the park, all of the major plant groups found in the roe deer diet (i.e., *Rubus* spp., dwarf shrubs and forbs) (Latham et al., 1999; Obidziński et al., 2013; Tixxier & Duncan, 1996) will be negatively impacted by the red deer population. It has been found that high white‐tailed deer (*Odocoileus virginianus*) density may lead to homogenization of vegetation associations and an increase in the graminoid share in the plant community (Rooney, 2009). Therefore, it is also predicted (P 6) that the high red deer pressure on vegetation will lead to an increase in the relative biomass of grasses, while the situation will be the opposite for the other plant groups.

## 2 METHODS

### 2.1 Study area

**FIGURE 1:**
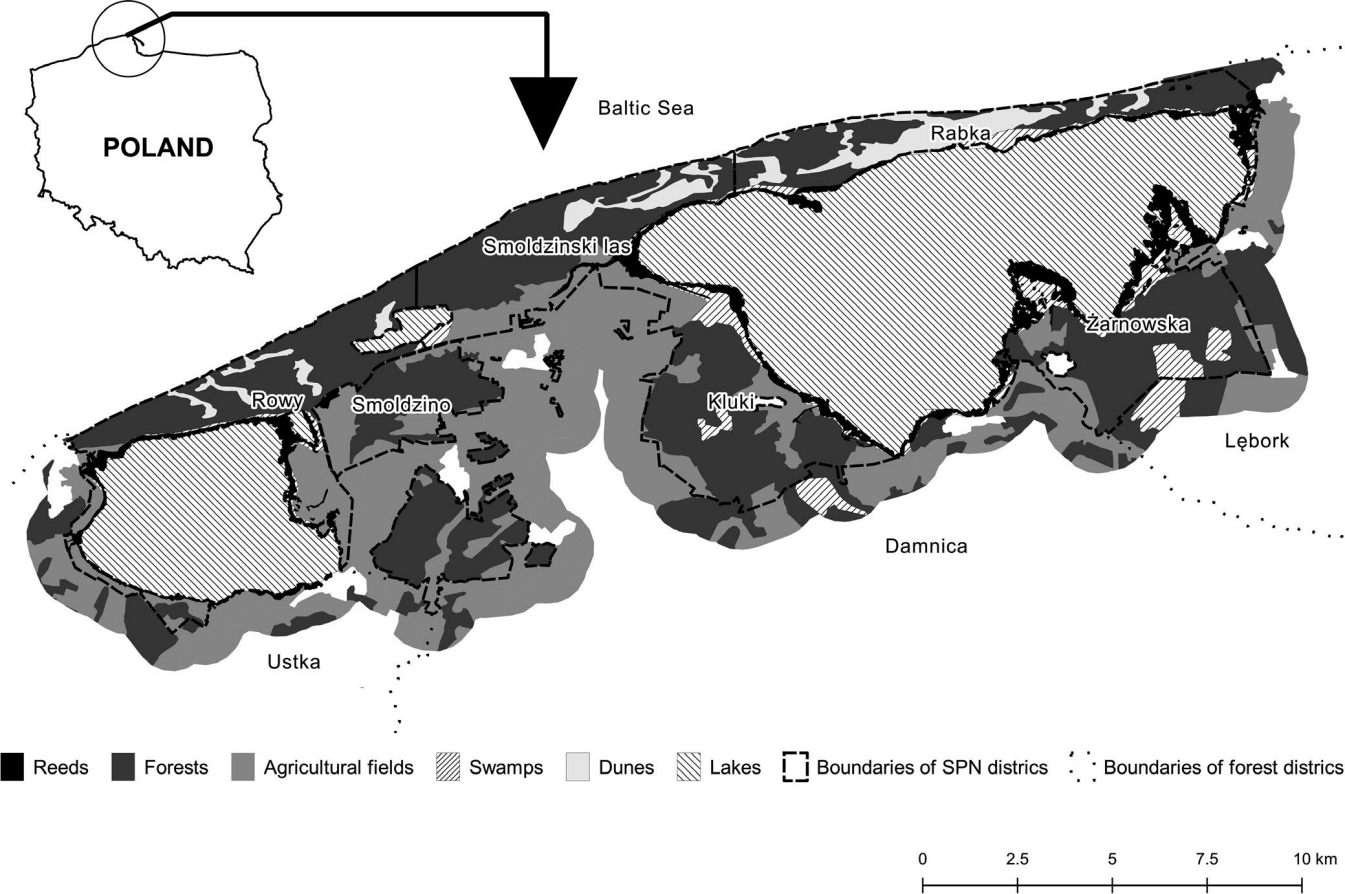
Location of the study area in Poland and a map of the habitat types in the Słowiński National Park and within a zone of 1 km in the neighboring forest districts (Ustka, Damnica, and Lębork)

The study was conducted in the Słowiński National Park (SNP) and in the adjoining two managed forest districts. The Słowiński National Park is a World Biosphere Reserve situated at the Baltic coast. The total area of the park is 32,700 ha. Land, sea, and freshwater lakes occupy 11,300 ha, 11,200 ha, and 10,300, respectively (Figure 1). Thus, about 66% of the land is covered by either freshwater or seawater. The freshwater area consists mainly of two lakes: Lake Łebsko (7,100 ha) and Lake Gardno (2,500 ha). Another 4,800 ha is covered by nonforest sites, of which a considerable share (1,900 ha) includes agricultural areas, such as mowed meadows and, to a lesser extent, arable grounds. The other nonforest sites comprise lake reeds and swamps (partly with forest cover). In these two site types, the groundwater level is high, which is why they are (to a large extent) inaccessible. Therefore, these sites provide favorable security cover for the red deer (Borkowski et al., 2019). In the nearest vicinity of the park, there is a variety of habitat types, including forest areas, meadows, and arable grounds (Figure 1). Most of the forests outside the park are owned by the state, whereas the majority of the agricultural areas belong to private owners.

Around 6,000 ha in the park are covered by forest sites, which are dominated (90%) by coniferous forest site types. The scots pine (*Pinus silvestris*) is the main tree species and it covers approximately 73% of the area. In the analysis of the park's forest sites, the same classification as the one used in Polish forest maps was applied (Table 1). This classification is used by state forest districts as well as by national parks. The Baltic Sea strongly influences the climate of the area, which means that winters are mild and summers rather cool and wet. The average yearly rainfall is approximately 700 mm.

**TABLE 1:** Coniferous forest habitat classification in Poland

Moisture groups of forest habitats	Fertility groups of forest habitats	
	Coniferous	Coniferous mixed
Dry	Coniferous dry (Cd)	Coniferous mixed dry (CMd)
Fresh	Coniferous fresh (Cf)	Coniferous mixed fresh (CMf)
Wet	Coniferous wet (Cw)	Coniferous mixed wet (CMw)
Swampy	Coniferous swampy (Cs)	Coniferous mixed swampy (CMs)

The ungulate community of the area is composed mainly of red and roe deer as well as the wild boar. Much less common and absent in the park is the fallow deer. As already noted, the average red deer density in the park is high and reaches approximately 26 ind./100 ha of the forest area (Borkowski et al., 2019) and is twofold to threefold lower in the surrounding forest districts. Hunting in the park is prohibited, while outside the park, all of the ungulate species are hunted with intensities of 1.5 ind./100 ha, 1.2 ind./100 ha, and 1.9 ind./100 ha (data per 100 ha of the hunting ground, including all the habitats present) of red deer, roe deer, and wild boar, respectively. Although wolves are also present in the SNP and in the forest districts, their precise numbers are unknown. In the southern part, the park borders three forest districts (Damnica, Lębork, and Ustka); however, the boundary with Ustka runs largely through Lake Gardno (Figure 1). The forest area of the districts ranges from 16,200 ha (Ustka) to 18,800 ha (Lębork). Since the share of the forests in the administration range of the districts varies from 25% (Damnica) to 35% (Lębork), the forests generally are rather scattered. The forest stands are also dominated by Scots pine (60%–65%). Numerous tourists visit the park mostly attracted by its extraordinary beaches, most intensively in summer (over 300 thousand people a year).

### 2.2 Pellet counts

The data were collected between 2015 and 2017. Pellet group counting (Alverson et al., 1988; Mayle et al., 1999), that is, fecal standing crop, was used to estimate the deer density and distribution. This method has been proven to provide reliable results concerning both deer density estimation (Camargo‐Sanabria & Mandujano, 2011; deCalesta, 2013) and habitat use (Borkowski, 2004; Borkowski & Ukalska, 2008). The pellet group counts were carried out only in the forest area because, as mentioned earlier, some of the nonforest sites are inaccessible.

In the Polish conditions, because the summer and autumn disappearance rate of pellet groups is rather high (Aulak & Babińska‐Werka, 1990), the data obtained by the pellet group counts are more reliable in winter. To estimate the deer density, November 15 was chosen as the beginning of the accumulation period. By that time, most of the tree leaves had fallen and the pellet groups were no longer covered by them. Since the pellet group counting usually lasted several days, the middle point of this period was assumed as the end of the pellet accumulation period. The roe deer defecation ratio for the density estimation of this species was assumed to be 20 (Mitchel et al., 1985), while the density of the red deer was taken from Borkowski et al. (2019).

Pellet groups were counted every year in the SNP (2015–2017) in the Damnica Forest district only in 2015 and in Lębork only in 2017. The counts were conducted in March along 2‐m‐wide transects. Since the transects were at first drawn on a map (only within forest areas), their distribution was roughly equal in the area. When walking in the field, the direction of the transects was kept with the aid of a compass. The data on red deer pellet group number were recorded for a 200‐m section of the transect, and the coordinates of the beginning and the end of a section were recorded using GPS receiver. Since the time of data collection was after the snow melted but before the beginning of vegetation season, the pellet detectability was not limited by the ground plants. As already mentioned, since the area of the Ustka Forest District was divided by Lake Gardno, pellet groups were counted only in two forest districts (Damnica and Lębork).

Deer pellet decay rates may differ in different climate and habitat (forest type, nonforest habitats) conditions; however, both areas (the SNP and the forest districts) are located close to each other. Moreover, the pellet groups were counted only in the forest habitats and pine is the dominating tree species in both areas. As a result, the differences in the pellet decay ratio were not considered a significant issue in this study. Deer pellets were differentiated by their size and shape. Red deer pellets are approximately twice larger than those of roe deer, while roe deer feces are rounder than red deer feces. Moreover, the pellet groups in this study were counted by two experienced fieldworkers; therefore, the possible misidentification of the deer species was probably marginal.

### 2.3 Roe deer food availability

Data on the deer pressure on forest vegetation were only collected in the SNP, and it was based on 106 fenced plots established in October 2014. The size of the plots was 7 × 7 m, and they were located in coniferous site types proportionally to the share of the sites in the park. The plots were distributed in transects (usually five plots in each). Each transect was located within the same forest site type, and the distance between the plots in a single transect was around 200 m. In September 2018, within each plot, three circular subplots (25 cm in diameter) were distinguished and all the plant biomass inside them was cut. The plants were divided into grasses, forbs, dwarf shrubs, and *Rubus* spp., and the dried biomass of the plant groups was measured. A similar protocol was applied for three circular subplots located near the plots. In total, data were collected from 318 subplots paired for fenced and unfenced plots. Tree seedlings and saplings, as well as lichens and mosses (which are seldom consumed by deer), were not considered in this study. The mean of relative share of each plant group was calculated separately in relation to the total plant biomass for the fenced and unfenced plots. Since the red deer density in the park was incomparably higher than both the roe deer and the wild boar, it was most likely the red deer that exerted the highest impact on the SNP vegetation.

### 2.4 Habitat features

The roe deer density was first analyzed depending on the forest site type (including its fertility and moisture). Using a digital map, each 200‐m section was matched with the forest site type. The distance between a 200‐m section and each type of the nearest nonforest habitats was then estimated.

### 2.5 Statistical analysis

The difference between roe and red deer pellet group densities in the Słowiński National Park and the difference between roe deer pellet group densities in the park and the managed forest districts were analyzed using the generalized Poisson log‐linear model with *p* < 0.05.

In the next stage, the roe deer pellet group density in the SNP forests was analyzed using an analysis of covariance (ANCOVA) for a generalized Poisson log‐linear model. The fixed effect of the model was a forest site type with the following covariates: red deer pellet group density, shortest distances to reed patch, meadow, swamp, arable ground, and tourist trail. The model was built using the best subset procedure, and the most informative model was selected based on the Akaike information criterion (AIC). The impact of the red deer on roe deer distribution was calculated using the mean number of red deer pellet groups on each 200‐m transect section per every category of roe deer pellet group numbers (0, 1, 2, …, 24). The relation between the red and roe deer distributions as well as the relations between the distances to the nonforest habitats and roe deer space use was analyzed using regression models.

The differences in herbaceous and dwarf shrub plant biomass between the fenced and unfenced plots as well as the differences in the relative share of the plant groups inside and outside the fenced plots were analyzed using a nonparametric Mann–Whitney *U* test with *p* < 0.05. All of the analyses were performed using Statistica 13.3 software (TIBCO Software Inc., 2017).

## 3 RESULTS

During the study, altogether there were 13,751 deer pellet groups counted, including 1,763 and 11,988 which belonged to the roe and red deer, respectively. In the park, there were 711 roe deer and 9, 252 red deer pellet groups recorded, while the respective numbers for the forest districts were 1,052 and 2,736. As predicted (P 1), the average roe deer pellet group density in the park was significantly lower than red deer pellet group density (Wald stat. = 15.27; *p* < 0.0001). The roe deer population density estimated for the SNP was between 1.3 and 3.6 ind./100 ha (of the forest area), depending on the year of the study (2.4 ind./100 ha on average). According to the prediction (P 2), the roe deer pellet group density in the park (grouped for all the years of the study) was lower than in Damnica (Wald stat. = 117.48; *p* < 0.0001) and in Lębork (Wald stat. = 108.72; *p* < 0.0001), while the values for both forest districts did not differ (Wald stat. = 0.03; *p* = 0.85). Roe deer densities estimated for Damnica and Lębork were 4.6 and 4.0 ind./100 ha of the forest area, respectively. Thus, based on the red deer density estimation in the park (26 ind./100 ha) and the commercial forest districts (Damnica—12.5 ind./100 ha and Lębork—10.9 ind./100 ha) (Borkowski et al., 2019), it was possible to calculate the red‐to‐roe deer density ratios in the areas. This ratio was 10.8 for the SNP and 2.7 for both forest districts.

**FIGURE 2:**
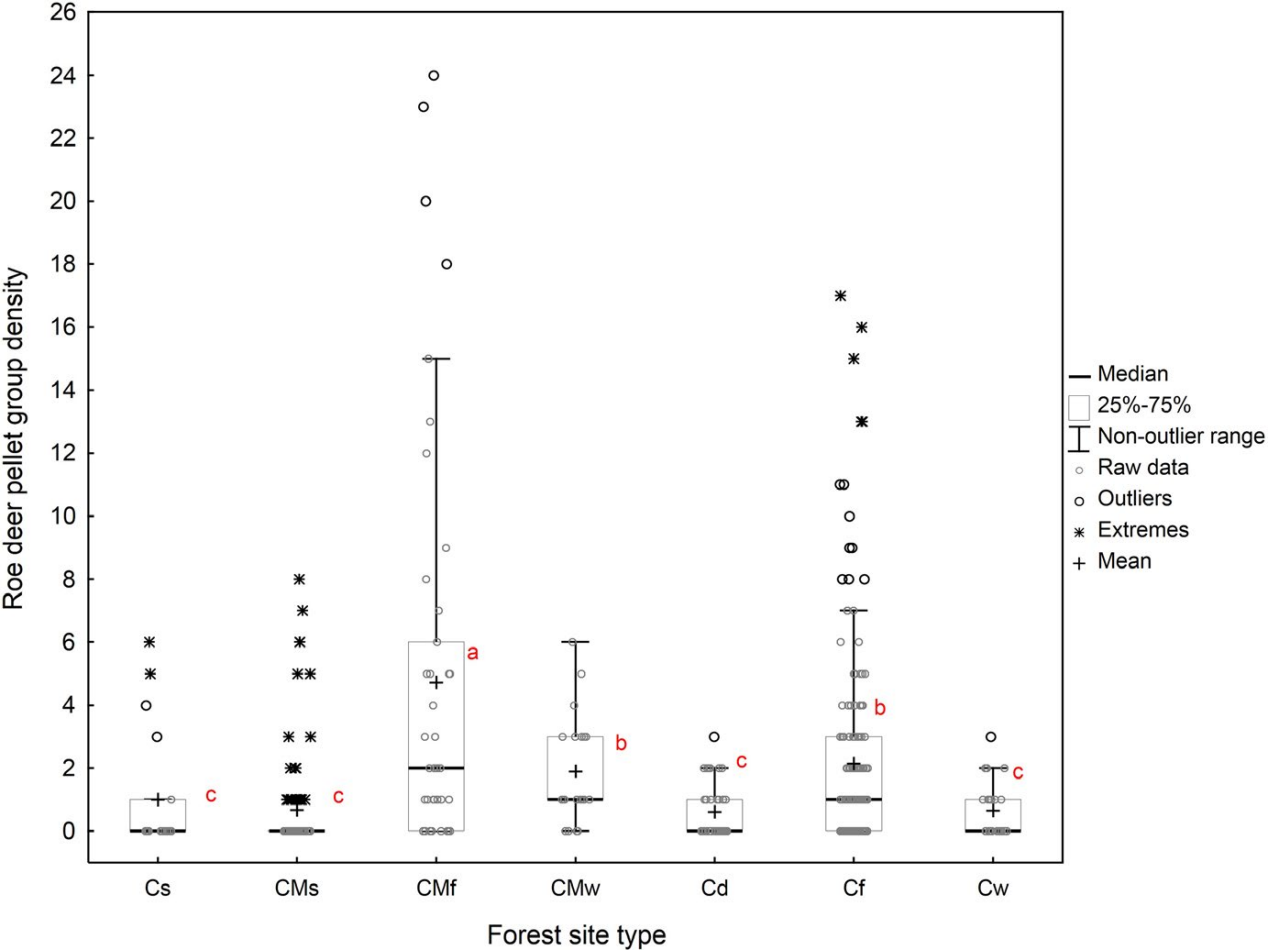
Roe deer pellet group density (per 200 m of transect) in the following forest site types: Cs, Coniferous Swampy; CMs, Coniferous Mixed Swampy; CMf, Coniferous Mixed Fresh; CMw, Coniferous Mixed Wet; Cd, Coniferous Dry; Cf, Coniferous Fresh; Cw, Coniferous Wet

**FIGURE 3:**
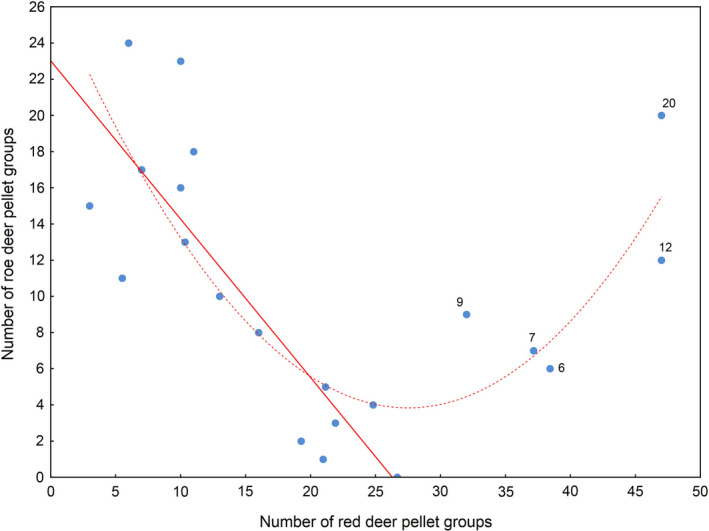
Average number of red deer pellet groups per 200‐meter transect for each category of roe deer pellet group numbers in the SNP. Numbers given next to the points over 30 red deer pellet groups represent the category of roe deer number pellet groups recorded alongside the respective 200 m transects

The roe deer pellet group density in the park was significantly affected by the forest site type, red deer pellet group density, distance to swamps, and distance to arable grounds (Table 2). The highest roe deer density was recorded in CMf (Figure 2), and this density was significantly lower in CMw and Cf. The lowest roe deer pellet group densities were noted in all the other forest site types, with no differences (*p* > 0.05) between them. Roe deer pellet group density was under the significant effect of red deer pellet group density (Figure 3). In general, the effect was curvilinear (up to approx. 30 pellet groups per 200 m transect), initially the red deer pellet group density negatively affected the roe deer pellet group density, but the effect later became positive. However, the number of cases with over 30 red deer pellet groups per 200‐m transect section was relatively very low. For the five cases found (Figure 3) which were responsible for the positive effect, the 32, 37, 38, 47, and 48 red deer pellet groups corresponded to the 9, 7, 6, 12, and 20 roe deer pellet groups, respectively. The number of 200‐m transect sections with over 30 red deer pellet groups, responsible for the positive effect, constituted just 4.6% (18 out of 395) of the total number transect sections. As a result, they were considered outliers and the linear relation was considered more reliable (Figure 3). Thus, the roe deer pellet group density in the park was highly significantly negatively (*r* = −0.84; *p* < 0.0001) affected by the red deer pellet group density. Moreover, in both forest districts, there was no correlation between red and roe deer pellet groups (Damnica *r* = 0.21 and Lębork *r* = 0.33; in both cases *p* > 0.05), which supports the prediction (P 3). The roe deer pellet group density in the forest area of the SNP was also significantly affected by the distance to two nonforest habitats (Tables 2 and 3): The relation with the distance to arable grounds was negative (*r* = −0.85, *p* < 0.0001), while that to swamps was positive (*r* = 0.80, *p* < 0.0001). Since the effects of the distance to meadows and reeds were not considered significant by the model, the prediction (P 4) was only partly sustained.

**TABLE 2:** GLM model (with the lowest Akaike's criterion) explaining roe deer pellet group density in the forests of the SNP

Effect	*df*	Wald statistics	Estimate	*p*‐value
Forest site type	6	61.06	‐	<0.0001
Red deer pellet group number	1	12.49	0.00896	0.0004
Distance to swamps	1	118.65	0.00048	<0.0001
Distance to arable grounds	1	63.04	−0.00017	<0.0001

**TABLE 3:** Relation between roe deer pellet group density and distance to nonforest habitats in the SNP

Distance to	Parameter	Parameter value	*p*	*R* ^2^	*r*
Arable grounds	*b*_0_	18.942	<0.0001		
	*b*_1_	−0.004	<0.0001	0.73	−0.85
Swamps	*b*_0_	−0.065	0.9754		
	*b*_1_	0.005	<0.0001	0.64	0.80

According to P 5, all of the major plant groups consumed by the roe deer as well as grasses in the fenced plots provided significantly higher amounts of biomass (for all groups *p* ≤ 0.0023) than in the unfenced plots (Table 4). The average amount of grass biomass was 28% higher in the fenced plots than in the unfenced plots. For the dwarf shrubs and forbs, the respective percentages were 42.7% and 81.8%, while in the case of *Rubus* spp. the biomass amount in the fenced plots was 349% higher than in the unfenced plots. Relative amounts of grass biomass did not differ (*p* > 0.05) between the fenced and unfenced plots (Table 5). A similar situation was recorded for forbs (*p* > 0.05). However, the relative amount of biomass of *Rubus* spp. was significantly higher (*p* = 0.0103) in the fenced plots than in the unfenced plots, while for the dwarf shrubs the trend was opposite (higher share in the unfenced plots) and approached a significant level (*p* = 0.0569). As a result, P 6 was rejected.

**TABLE 4:** Comparison of major plant group biomass within fenced (In) and unfenced (Out) plots in the SNP

Plant biomass	n/N	Raw data		Mean rank		p
		In	Out	In	Out	
Grasses	97/106	163 (24.9)	127 (21.5)	109	82	0.0023
*Rubus* spp.	34/106	291 (78.2)	64.8 (17.2)	45	22	<0.0001
Dwarf shrubs	88/106	287 (26.2)	201 (22.8)	101	76	0.0009
Forbs	80/106	48.2 (10.1)	26.5 (7.83)	89	59	0.0007

Abbreviations: *n*/*N*, number of twin‐fenced and unfenced plots per *N* total; mean (standard error of mean) of raw data; mean rank and *p*‐value Mann–Whitney *U* test.

**TABLE 5:** Mean share (%) of major plant groups in total biomass within fenced (In) and unfenced (Out) plots in the SNP

Share in total plant biomass	*n*/*N*	Raw data		Mean rank		*p*‐value
		In	Out	In	Out	
Grasses	97/106	30 (3)	33 (4)	99	92	0.5686
*Rubus* spp.	34/106	34 (5)	21 (4)	40	27	0.0103
Dwarf shrubs	88/106	65 (3)	68 (4)	81	96	0.0569
Forbs	80/106	10 (2)	10 (2)	82	66	0.1795

Note

*n*/*N*, number of twin‐fenced and unfenced plots per *N* total; mean (standard error of mean) of raw data; mean rank and *p*‐value Mann–Whitney *U* test.

## 4 DISCUSSION

Although the roe deer is not hunted in the Słowiński National Park, its density was lower than in the forest districts, where the populations are under hunting pressure. Therefore, there must be some factor(s) keeping the roe deer population in the park at a low level and its intensity must exceed the mortality generated by the hunters (and by other sources) outside the park. Factors causing roe deer mortality may be divided into two major groups: neonatal and subadult/adult mortality. The former mainly includes fox predation (Panzacchi et al., 2009), mowing (Jarnemo, 2002), or starvation/hypothermia in the first period of fawns’ lives (Jarnemo, 2002), while the most important in the latter are harvesting (Langvatn & Loison, 1999), predation by lynx (Andrén & Liberg, 2015) and/or wolves (Nowak et al., 2011), winter starvation (Aguirre et al., 1999), traffic accidents (Madsen et al., 2002), or poaching (Sönnichsen et al., 2017). Probably none of these, however, are specific to the park area and some of them (besides hunting) are probably more influential beyond its boundaries (poaching, traffic accidents). Due to presence of the meadows in the SNP, it is worth mentioning that mowing is also most probably not a more important source of roe deer neonatal mortality in the SNP than outside its boundaries, since in the park (due to the bird nesting season) mowing is done not earlier than 15 July (Andrzej Wróbel, Chief Forester, pers. comm.). The majority of the fawns are born at the end of May and beginning of June, while mowing is the most dangerous to the fawns in their first month of life (Jarnemo, 2002).

In a study comparing cervid density in two relatively large national parks in Germany and the Czech Republic, roe deer numbers were also higher out of the protected areas than inside them (Heurich et al., 2015). Likely reasons for this were the lack of winter feeding inside the parks, in combination with higher snow cover (the study was done in mountainous areas) as well as forest cover and lynx density exceeding those outside the parks. In the current study area, due to proximity of the Baltic Sea, the winters are rather mild (especially during last few years) and there is not much difference in the forest cover in the SNP and neighboring forest districts and lynx are absent in the region. However, it should also be noted that when the wolves are relatively rare, they prey upon roe deer more readily than on the much larger red deer (Nowak et al., 2011). However, the packs recorded in the SNP are relatively large (6–9 ind.; Andrzej Wróbel, Chief Forester, pers. comm.) and it seems improbable that with such a high red deer density as in the park the wolves would have selected roe deer (especially when its density is so low). Moreover, the park rangers regularly encounter red deer carcasses, most probably killed by the wolves.

Thus, competition with the red deer seems to be the most likely reason for the low roe deer density in the park. First of all, as mentioned, in the forest districts, where red deer densities were remarkably lower than in the park, roe deer densities exceeded those in the SNP. Secondly, which is equally important, the roe deer distribution in the SNP forest habitats was under the highly negative influence of the red deer density. Most probably, the impact was due to the red deer pressure on the vegetation (resource competition). In terms of the general plant biomass, the pressure was similar in all the forest site types and proportional to the plant abundance—in all the forest types, it was around 40% of the total plant biomass (Borkowski et al., 2019). However, it is most likely that the nutritional value of the remaining 60% is not the same as that of the 40%, since the plant parts of the highest quality (with lower fiber content) are eaten first (Naughton‐Treves et al., 2005). In general, according to reviews across Europe (Gebert & Verheyden‐Tixier, 2001; Tixxier & Duncan, 1996), the most important difference in the diet of both deer species is that graminoids predominate in the red deer, while in the case of the roe deer, as briefly mentioned earlier, the predominating element of its diet is *Rubus spp*. In addition, as already noted, forbs are an important part of the roe deer diet, especially in winter (Latham et al., 1999; Obidziński et al., 2013), whereas dwarf shrubs are selected by both species (Obidziński et al., 2013). In the present study, the biomass of all of the major vegetation groups (including those the most important in roe deer diet) was under the highly significant impact of red deer pressure (due to the low roe deer numbers in the park, it was assumed that the impact on vegetation of the species was negligible). As mentioned before, the red deer (as the more general feeder) diet range is wider and it includes all the food items found in the roe deer diet (Latham et al., 1999; Storms et al., 2008), although the red deer do not necessarily rely on them. As a result, when the red deer density is high, it is expected to have a negative impact on the roe deer forage availability and, consequently, on its populations.

This mechanism of competition for food was proposed to explain the negative influence of the red deer density on roe deer fawn body mass during the first winter of their lives (Richard et al., 2010). Moreover, the current data on spatial distribution were collected in winter, when the competition between both species is especially likely (Latham et al., 1999). In addition, it needs to be emphasized that in the forest districts adjoining the SNP, where the red deer densities were clearly lower, there was no relation between the red deer density and the roe deer distribution. Similarly, Borkowski and Ukalska (2008) found that in the conditions of much lower red deer density (4–6 ind./100 ha) than in the park, there was no influence of the red deer distribution on that of the roe deer. Thus, most probably, the existence of an interspecific negative influence of the red deer presence on roe deer population ecology and performance (small population size) is related to the red deer density. This is in accordance with the general knowledge of herbivore ecology, that is, an increase in the densities of species contributes to their competitive interactions (Owen‐Smith, 2010). Roe deer are 4–5 times smaller than red deer, and therefore, red deer food requirements (in terms of quantity) are accordingly higher. Thus, when red deer are ten times more numerous than roe deer (as in the SNP), their impact on vegetation (including the species important for the roe deer) must be much higher. The relationship between density and habitat quality is clearly also important. At the south‐western edge of both species’ range (Portugal), red deer density negatively influenced roe deer distribution even with a low population density of the former (2.3–4.7 ind./100 ha) (Torres et al., 2012).

Taking into account the high red deer density in the SNP, it is very probable (although the issue was not covered in this study) that the interference competition may also contribute to the negative influence of the red deer on the roe deer distribution in the park. Behavioral interference between the high‐density fallow deer population and the roe deer population was documented in Italy (Ferretti et al., 2008, 2011). The above‐mentioned studies demonstrated that the roe deer avoided the fallow deer at feeding sites or they were displaced due to the fallow deer aggression. In a study by Ferretti et al. (2015) on the effects of red deer on chamoix (*Rupicapra rupicapra*), besides behavioral interactions, the chamoix habitats were also negatively affected by red deer trampling and this could also be the case in the SNP.

No evidence was found that the high deer pressure on vegetation in the park leads to plant community homogenization. As mentioned earlier, in a 16‐year study, Rooney (2009) found that the impact of high white‐tailed deer density on the forest ground cover had promoted grasses and sedges. In the present research, the effects of deer pressure on the forest vegetation have been studied within a much shorter period (four years). It is, therefore, very probable that the period was too short to capture the trend. Nevertheless, it should be noted that even though the differences in the grass biomass within the fenced plots and outside of them were insignificant, in all the forest site types of the park the relative amount of biomass (in comparison with the other plant groups) of the fenced grasses was higher than that in the unfenced grasses (Borkowski et al., unpublished data). This issue requires further consideration using a longer data collection period.

Protected area management does not always meet the identified conservation priorities (van Beeck Calkoen et al., 2020; Chape et al., 2005). In Europe, since hunting is the most important source of ungulate mortality (Cromsigt et al., 2013) and the impact of predation on ungulate populations (especially in productive environments) may be limited (Melis et al., 2009), their (especially deer) densities in unhunted protected areas often tend to increase (Demarais et al., 2012). The current study demonstrates that high ungulate density (or at least some species within the assemblage), besides the negative impact on natural resources (Borkowski et al., 2019), may contribute to interspecific competition. In consequence, the high density of some species (probably, first of all, the general feeders) may lead to a decrease in the number of other species (more specialized ones).

## 5 CONCLUSION

According to the results of this study, the high red deer density may negatively affect the roe deer population number and spatial distribution. First of all, in the park, where the red deer density was high, despite the lack of hunting, the roe deer density was lower than in the hunted forest districts where, in turn, red deer populations were of remarkably lower numbers. Moreover, in the park's forests, both roe deer density and spatial distribution were under the negative influence of the red deer density, whereas in the adjacent forest districts, the trend was not recorded. Although behavioral interferences between the species cannot be excluded, the primary impact of the red deer on the roe deer population in the park was probably due to the pressure on vegetation. The red deer in the SNP exerted a significant influence on the plant availability, including those plant groups which form the basic part of the roe deer diet (*Rubus* spp., forbs).

Unhunted protected areas with productive environments (limited impact of predators on ungulate populations) may be especially prone to sustaining high densities of ungulate species, and through interspecific competition, some ungulate species may have much higher densities than others. In Europe, roe deer seems to be an inferior competitor to other species (e.g., fallow deer—*see Introduction*). The current study demonstrates that red deer may also outcompete the roe deer populations. If it takes place in the national parks, it may contradict one of their objectives, that is, to maintain viable and ecologically functional populations (Dudley, 2008). If the human intervention in the national parks is to be limited, ungulate populations in the protected areas should ideally be shaped by natural regulation (Sinclair, 1998). However, as it is shown in this study, such an approach may favor some species on account of others, which may hamper reconstructing ungulate assemblages in the protected areas (Venter et al., 2014). Modern wildlife management should ensure the long‐term viability and persistence of the ungulates as well as their predators (Apollonio et al., 2017). This is especially important in the case of specialized predators, dependent largely on one prey. An example of such a predator species is the Eurasian lynx (*Lynx lynx*), whose density tends to be related to the roe deer availability (Sidorovich, 2006). According to the wolf and lynx monitoring in Poland (GIOŚ, 2020), wolf conservation status is much more satisfactory than the lynx status and the conservation status of the latter in all eight monitoring sites throughout the country is unfavorably bad (U2). One of the reasons for this may be a decrease in the roe deer population numbers in many areas of Poland (Śmietana et al., unpubl. data). The potential role of the red deer in this depletion should also be assessed.

## CONFLICT OF INTEREST

None declared.

## AUTHOR CONTRIBUTION

**Jakub Borkowski:** Conceptualization (lead); Data curation (lead); Investigation (equal); Methodology (lead); Project administration (lead); Supervision (lead); Validation (lead); Writing‐original draft (lead); Writing‐review & editing (lead). **Rafał Banul:** Data curation (supporting); Investigation (supporting); Writing‐original draft (supporting); Writing‐review & editing (supporting). **Jola Jurkiewicz:** Data curation (supporting); Investigation (supporting); Writing‐original draft (supporting); Writing‐review & editing (supporting). **Czesław Hołdyński:** Conceptualization (supporting); Data curation (supporting); Investigation (supporting); Methodology (supporting); Writing‐original draft (supporting); Writing‐review & editing (supporting). **Justna Święczkowska:** Data curation (supporting); Investigation (supporting); Writing‐original draft (supporting); Writing‐review & editing (supporting). **Maciej Nasiadko:** Data curation (supporting); Formal analysis (supporting); Investigation (supporting); Software (supporting); Visualization (supporting); Writing‐original draft (supporting); Writing‐review & editing (supporting). **Dariusz Załuski:** Conceptualization (supporting); Data curation (supporting); Formal analysis (lead); Investigation (supporting); Software (lead); Validation (supporting); Visualization (lead); Writing‐original draft (supporting); Writing‐review & editing (supporting).

## Data Availability

All necessary data for this study are available on the Dryad digital repository—https://doi.org/10.5061/dryad.95x69p8k3.
